# Near full length genome of a recombinant (E/D) cosavirus strain from a rural area in the central region of Brazil

**DOI:** 10.1038/s41598-018-30214-1

**Published:** 2018-08-17

**Authors:** Antonio Charlys da Costa, Adriana Luchs, Flavio Augusto de Pádua Milagres, Shirley Vasconcelos Komninakis, Danielle Elise Gill, Márcia Cristina Alves Brito Sayão Lobato, Rafael Brustulin, Rogério Togisaki das Chagas, Maria de Fátima Neves dos Santos Abrão, Cassia Vitória de Deus Alves Soares, Xutao Deng, Ester Cerdeira Sabino, Eric Delwart, Élcio Leal

**Affiliations:** 1 Institute of Tropical Medicine, University of São Paulo, São Paulo, Brazil; 2 Enteric Disease Laboratory, Virology Center, Adolfo Lutz Institute, São Paulo, Brazil; 3 LIM/46, Faculty of Medicine, University of São Paulo, São Paulo, Brazil; 4 Secretary of Health of Tocantins, Tocantins, Brazil; 5 Federal University of Tocantins, Tocantins, Brazil; 6 Public Health Laboratory of Tocantins State (LACEN/TO), Tocantins, Brazil; 7 Postgraduate Program in Health Science, Faculty of Medicine of ABC, Santo André, Brazil; 8 Retrovirology Laboratory, Federal University of São Paulo, São Paulo, São Paulo Brazil; 9 Blood Systems Research Institute, San Francisco, USA; 10 Department Laboratory Medicine, University of California San Francisco, San Francisco, USA; 11 Institute of Biological Sciences, Federal University of Pará, Pará, Brazil

## Abstract

In the present article we report the nearly full length genome of a Cosavirus strain (BRTO-83) isolated from a child with acute gastroenteritis, and who is an inhabitant of a rural area in the central region of Brazil. The sample was previously screened and negative for both: common enteric viruses (i.e. rotavirus and norovirus), bacteria, endoparasites and helminthes. Evolutionary analysis and phylogenetic inferences indicated that the Brazilian BRTO-83 Cosavirus strain was a recombinant virus highly related to the E/D recombinant NG385 strain (Genbank JN867757), which was isolated in Nigeria from an acute flaccid paralysis patient. This is the first report of a recombinant E/D Cosavirus strain detected in Brazil, and the second genome described worldwide. Further surveillance and molecular studies are required to fully understand the epidemiology, distribution and evolution of the Cosavirus.

## Introduction

The family *Picornaviridae* has undergone a significant expansion in recent years, due principally to the identification of previously unknown picornaviruses by next-generation sequencing (NGS) of clinical and environmental samples. The family is divided currently into 29 genera, and the natural hosts of these viruses are vertebrates, including mammals and birds. Enteroviruses, polioviruses, hepatoviruses and aphthoviruses are the most exhaustively characterized animal pathogens, and are associated with a wide spectrum of clinical manifestations, including undifferentiated febrile illness, respiratory illness, aseptic meningitis and acute flaccid paralysis (AFP)^1^. Recently, metagenomics analysis made possible the identification of three new human picornaviruses: klassevirus, salivirus, and cosavirus^2–4^. The pathogenicity of Cosaviruses in humans remains unknown. However, Cosaviruses have been detected in human fecal specimens, and studies have suggeed its association with gastroenteritis infection^5,6^. The genus Cosavirus consists of five genotypes (Cosavirus A, Cosavirus B, Cosavirus D, Cosavirus E and Cosavirus F) and is most closely related to members of the Cardiovirus and Senecavirus genera^2,7^.

We herein report the detection of a recombinant Cosavirus strain (BRTO-83) isolated from a fecal sample obtained from a 1-year-old-child patient presenting acute gastroenteritis in 2013, in the state of Tocantins, Central Brazil. Tocantins is characterized by dry grassland, scrub forests, vast rivers and soybean plantations. The modern capital, Palmas, was purposefully built in the geographic center of the state and is surrounded by forested hills. The patient was an inhabitant of Barra do Ouro, a small municipality (4,460 inhabitants) located 422 km from the state capital, Palmas.

## Results and Discussion

A survey conducted throughout in the northern state of Tocantins in Brazil was performed during the years 2010 through 2016 to screen human feces for the presence of enteric pathogens. Fecal specimens were screened for enteric viruses (i.e. rotavirus and norovirus), bacteria (i.e *E*. *coli* and *Salmonella sp*.), endoparasites (i.e. *Giardia sp*.) and helminthes using conventional (i.e. culture techniques) and molecular methods (i.e. commercial enzyme immunoassays). NGS techniques were used to identify possible unscreened or undetected enteric viruses, and a recombinant Cosavirus strain was detected in sample BRTO-83.

**Figure 1:**
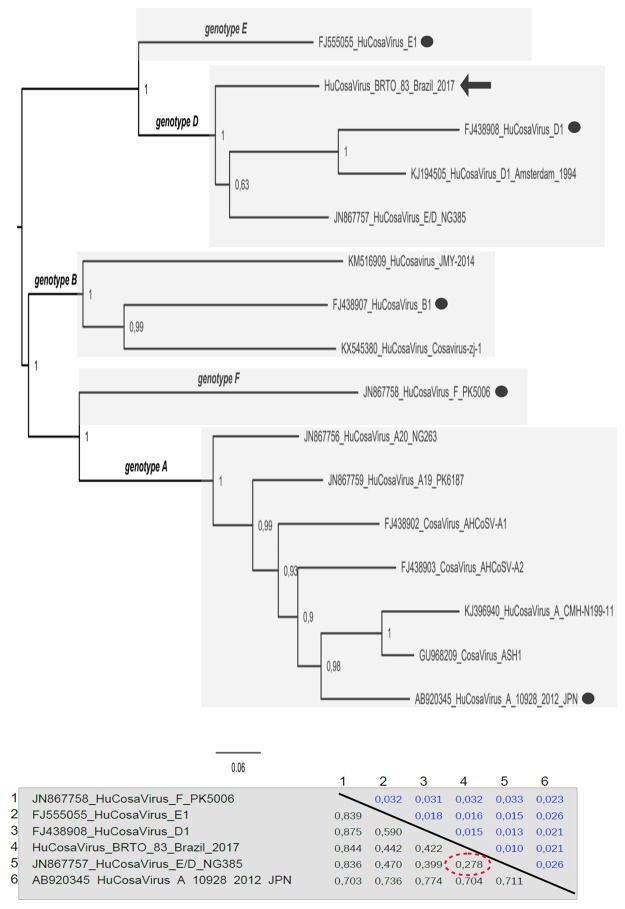
Phylogenetic tree constructed using near-full-length genomes of the human cosaviruses. The Maximum likelihood tree was constructed with genomes of human strains of cosaviruses (HuCosaVirus). The Brazilian strain BRTO-83, described in this study, is indicated by a filled arrow. The values on the tree indicate the statistical support of each node that was calculated using the Shimodaira-Hasegawa test. Genotype reference strains are indicated by filled dots on the genome tree. The panel in the figure is a matrix that shows the values of genetic distances between some isolates. Values below the diagonal are genetic distances and values above the diagonal are standard errors. These distances were estimated using Mega software version 7.0.

**Figure 2:**
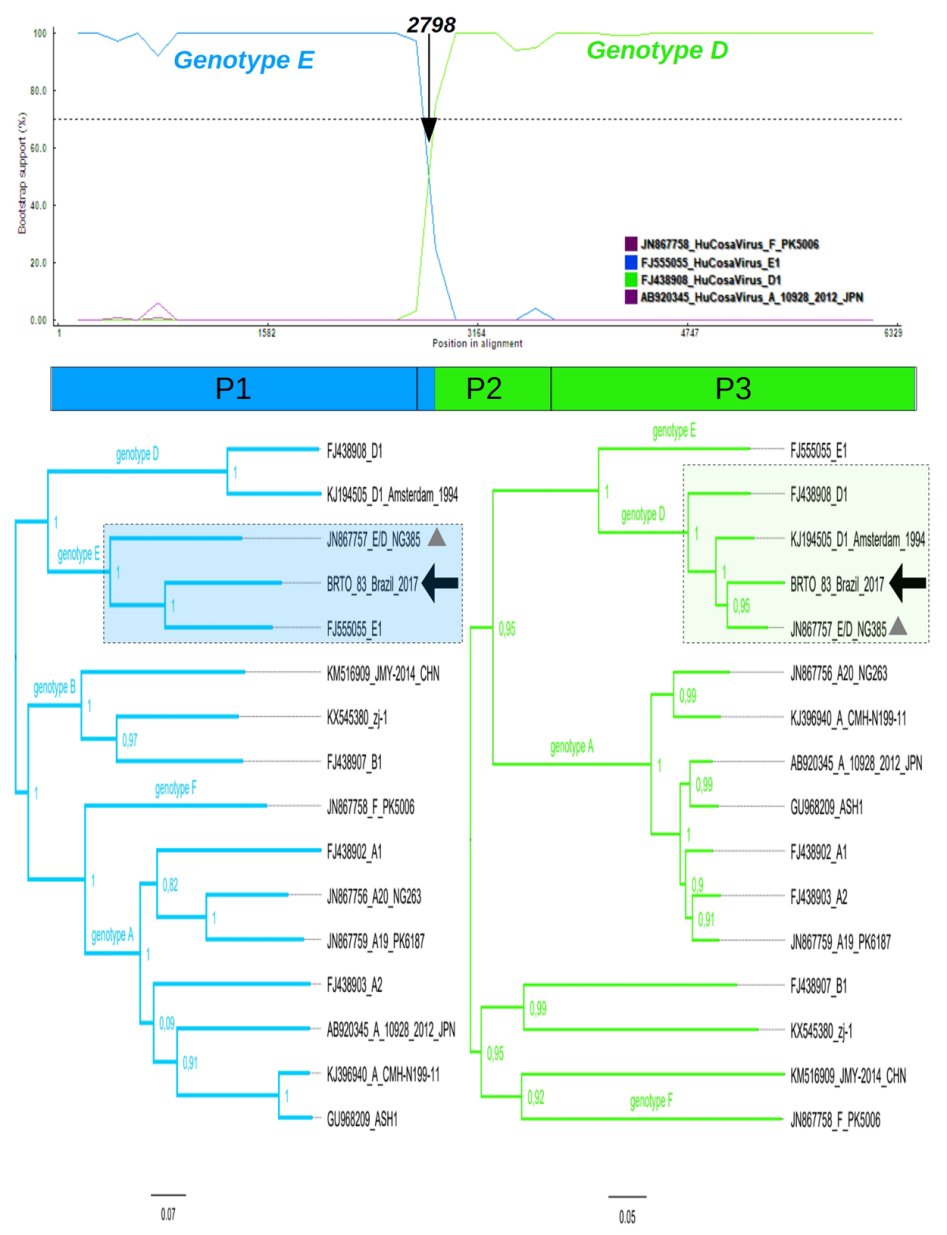
Recombination pattern of the Brazilian isolate BRTO-83. Colored lines represent the probability (given in bootstrap value) that genomic regions belong to a certain parental genotype. The x-axis represents the sequence length in base pairs (bp). The y-axis represents the statistical support (bootstrap) based on 500 replicates. Each plotted line refers to a certain genotype (see the code color on the small panel). A vertical arrow indicates the recombination break point on the genome region. The plot indicates a single breakpoint in the polyprotein region of the isolate BRTO-83 at the position 2798. This breakpoint corresponds to the initial part of the Cosavirus protein P1 (see diagram below the recombination plot). Strains used as parental references are also shown in the recombination plot. Trees were constructed using the partitioned genome. The first partition tree (nucleotides; 1–2798) demonstrates the isolate BRTO-83 clustering with genotype E (left tree), and the second tree (nucleotides 2799–6269) demonstates the isolate BRTO-83 within the clade formed by genotype D strains (right tree). Genotype reference strains are indicated by filled dots on the genome tree. The recombinant strain NG385 is indicated by gray triangles in the trees. Analyses were performed using a neighbor-joining method and a Kimura 2 parameters model in windows of 250 bp sliding along sequences in increments of 40 bp. For parental references, non-recombinant sequences were used. The boostscan plot was obtained by analysis performed using RDP v.4 software.

The near full length genome (6278 nt) was sequenced and compared with previously described Cosavirus strains (Fig. 1 and Supplementary data). Recombination analysis indicated that the first 2680 bases of the BRTO-83 genome are related to the genotype E, while the remaining nucleotides are related to the genotype D (Fig. 2 and Supplementary data). This pattern of Cosavirus recombination has been previously reported only once, in 2007, from a Nigerian patient suffering from AFP (NG385 strain; JN867757). Additional phylogenetic analysis, performed using complete genomes and the partitions corresponding to the genome breakpoints, confirmed that the Brazilian BRTO-83 strain was closely related to the NG385 strain E/D from Nigeria. The recombination breakpoint detected in both isolates, BRTO-83 and NG385, coincided, suggesting that both sequences display identical mosaic patterns (Fig. 2 and Supplementary Material). The inferred trees, which were assembled using the partitions and composed by free recombination regions also suggest that the recombinant E/D strains detected in Brazil and Nigeria may share the same evolutionary ancestor (Fig. 1 and Supplementary data).

The child infected by the BRTO-83 strain was suffering from a severe episode of diarrhea, and the data strongly suggests that a Cosavirus was, in fact, the causative agent of the disease. A common feature of virome analysis is that the epidemiological background information on potential contact with animals, consumption of contaminated food and/or medical records of the patients is not generally available. The present study is no exception; there was no epidemiological connection between the patient and the virus. Cosaviruses are not pathogens recognized to be associated with gastroenteritis, and the patient may have been infected with other bacteria and parasites not recognized in the screening flow. In addition, the protocol applied focused on viral metagenomics and removed eukaryotic cells from the test samples, making impossible the use of the BRATO-83 sample library to screen for reads from bacteria, parasites or helminthes.

Human Cosavirus infection reports are still very limited worldwide, but the association of the viruses with gastroenteritis is increasing^4,8,9^. In Brazil, there is only one study considering Cosaviruses role; nevertheless its association with gastroenteritis could not be established^3^. This is the first report of a recombinant E/D Cosavirus strain detected in Brazil, and the second genome described worldwide. Further surveillance and molecular studies are required to fully understand the epidemiology, distribution and evolution of the Cosavirus.

## Materials and Methods

The protocol used to perform deep-sequencing was a combination of several protocols normally applied to viral metagenomics and/or virus discovery, and has been partially described by da Costa *et al*.^10^. In summary, 50 mg of the human BRATO-83 fecal sample was diluted in 500 μl of Hanks’ buffered salt solution (HBSS), added to a 2 ml impact-resistant tube containing lysing matrix C (MP Biomedicals, USA) and homogenized in a FastPrep-24 5G Homogenizer (MP biomedicals, USA). The homogenized sample was centrifuged at 12,000 × *g* for 10 min, and approximately 300 μl of the supernatant was then percolated through a 0.45 μm filter (Merck Millipore, Billerica, MA, USA) in order to remove eukaryotic- and bacterial- cell-sized particles. Approximately 100 μl, roughly equivalent to one fourth of the volume of the tube of cold PEG-it Virus Precipitation Solution (System Biosciences, CA, USA), was added to the obtained filtrate, and the contents of the tube were gently mixed then incubated at 4 °C for 24 hours. After the incubation period, the mixture was centrifuged at 10000 × *g* for 30 minutes at 4 °C. Following centrifugation, the supernatant (~350 μl) was discarded. The pellet, rich in viral particles was treated with a combination of nuclease enzymes (TURBO DNase and RNase Cocktail Enzyme Mix - Thermo Fischer Scientific, CA, USA; Baseline-ZERO DNase - Epicentre, WI, USA; Benzonase - Darmstadt, Germany; and RQ1 RNase-Free DNase and RNase A Solution - Promega, WI, USA) in order to digest unprotected nucleic acids. The resulting mixture was subsequently incubated at 37 °C for 2 h.

After incubation, viral nucleic acids were extracted using a ZR & ZR-96 Viral DNA/RNA Kit (Zymo Research, CA, USA) according to the manufacturer’s protocol. The cDNA synthesis was performed with AMV reverse transcription (Promega, WI, USA). A second strand of cDNA synthesis was performed using DNA Polymerase I Large (Klenow) Fragment (Promega, WI, USA). Subsequently, a Nextera XT Sample Preparation Kit (Illumina, CA, USA) was used to construct a DNA library, which was identified using dual barcodes. For size range, Pippin Prep (Sage Science, Inc.) was used to select a 300 bp insert (range 200–400 bp). The library was deep-sequenced using the Hi-Seq 2500 Sequencer (Illumina, CA, USA) with 126 bp ends. Bioinformatic analysis was performed according to the protocol previously described by Deng *et al*.^11^. Contigs sharing a percent nucleotide identity of 95% or less were assembled from the obtained sequence reads by *de novo* assembly. The contigs included the rotavirus sequences, as well as others, such as enteric viruses, human, fungal and bacterial sequences. The resulting singlets and contigs were analyzed using BLASTx to search for similarity to viral proteins in GenBank’s Virus RefSeq. The contigs were compared to the GenBank nonredundant nucleotide and protein database (BLASTn and BLASTx). After the identification of a Cosavirus, a reference template sequence was used for mapping the full-length genome with Geneious R9^7^.

BLASTn was initially used to identify viral sequences through their sequence similarity to annotated viral genomes in GenBank. Based on the best hits of the blastx searches, the following 14 polyprotein (6329 nt) sequences, listed by their Genbank numbers, were choosen to be used in the next analyses: JN867756, JN867757, JN867758, JN867759, FJ555055, FJ438908, KJ194505, KM516909, AB920345, KJ396940, GU968209, FJ438903, FJ438902, FJ438907 and KX545380. These genomes were then aligned using Clustal × software^12^. Subsequently a phylogenetic tree was constructed by the Maximum Likelihood approach and branch support values were assessed by the Shimodaira-Hasegawa test. All trees were inferred using PhyML software^13^. The KHY model and gamma distribution were selected according to the likelihood ratio test (LRT) implemented in the jModeltest software^14^.

To determine the extent of recombination among sequences, we used software RDP v.4, which utilizes a collection of methods. An excellent and detailed explanation of each method implemented in the RDP software can be found in the user’s manual (http://darwin.uvigo.es/rdp/rdp.html). Breakpoints were identified by means of software Simplot, version 2.5, which is available at http://sray.med.som.jhmi.edu/RaySoft. Recombination was checked by means of Bootscan, using the neighbor-joining approach and the F84 substitution model. A sliding window of 350 bp with 60-bp increments was used. Window trees were replicated 300 times to provide bootstrap support for permuted trees.

### Accession number

The BRTO-83 strain has been deposited in GenBank under accession number MG979713.

### Ethical approval

Previous Ethics Committee approval was granted by the Adolfo Lutz Institute, São Paulo, Brazil (CEP 965.723; CTC 45G-2014), the Faculdade de Medicina da Universidade de São Paulo (CAAE: 53153916.7.0000.0065) and the Centro Universitário Luterano de Palmas - ULBRA (CAAE 53153916.7.3007.5516). This was an anonymous unlinked study and informed consent was not required according to resolution 466/12 concerning research involving humans (Conselho Nacional de Saúde/Ministério da Saúde, Brasília, 2012).

## Electronic supplementary material

Supplementary Information
