# Supplementary material for: Near full length genome of a recombinant (E/D) cosavirus strain from a rural area in the central region of Brazil
Source: Sci Rep. 2018 Aug 17;8:12304. doi: 10.1038/s41598-018-30214-1 (PMC6098101; doi:10.1038/s41598-018-30214-1)
Supplement: Supplementary file 1 — Supplementary Information [file 41598_2018_30214_MOESM1_ESM.pdf]

Near full length genome of a recombinant (E/D) cosavirus strain from a rural area in the central region of Brazil

Antonio Charlys da Costa[1], Adriana Luchs[2], Flavio Augusto de Pádua Milagres[3,4,5,6], Shirley Vasconcelos Komninakis[7,8], Danielle Elise Gill[1], Márcia Cristina Alves Brito Sayão Lobato[4,6], Rafael Brustulin[4,5,6], Rogério Togisaki das Chagas[4,6], Maria de Fátima Neves dos Santos Abrão[4,6], Cassia Vitória de Deus Alves Soares[4,6], Xutao Deng[9,10], Ester Cerdeira Sabino[1,3], Eric Delwart[9,10], Élcio Leal[11]

1 Institute of Tropical Medicine, University of São Paulo, São Paulo, Brazil
2 Enteric Disease Laboratory, Virology Center, Adolfo Lutz Institute, São Paulo, Brazil
3 LIM/46, Faculty of Medicine, University of São Paulo, Brazil
4 Secretary of Health of Tocantins, Tocantins, Brazil
5 Federal University of Tocantins, Tocantins, Brazil
6 Public Health Laboratory of Tocantins State (LACEN/TO)
7 Postgraduate Program in Health Science, Faculty of Medicine of ABC, Santo André, Brazil
8 Retrovirology Laboratory, Federal University of São Paulo, São Paulo, São Paulo, Brazil
9 Blood Systems Research Institute, San Francisco, USA
10 Department Laboratory Medicine, University of California San Francisco, San Francisco, USA.
11 Institute of Biological Sciences, Federal University of Pará, Pará, Brazil.

Supplementary material:
Locations of the Breakpoint in the genome HuCosaVirus

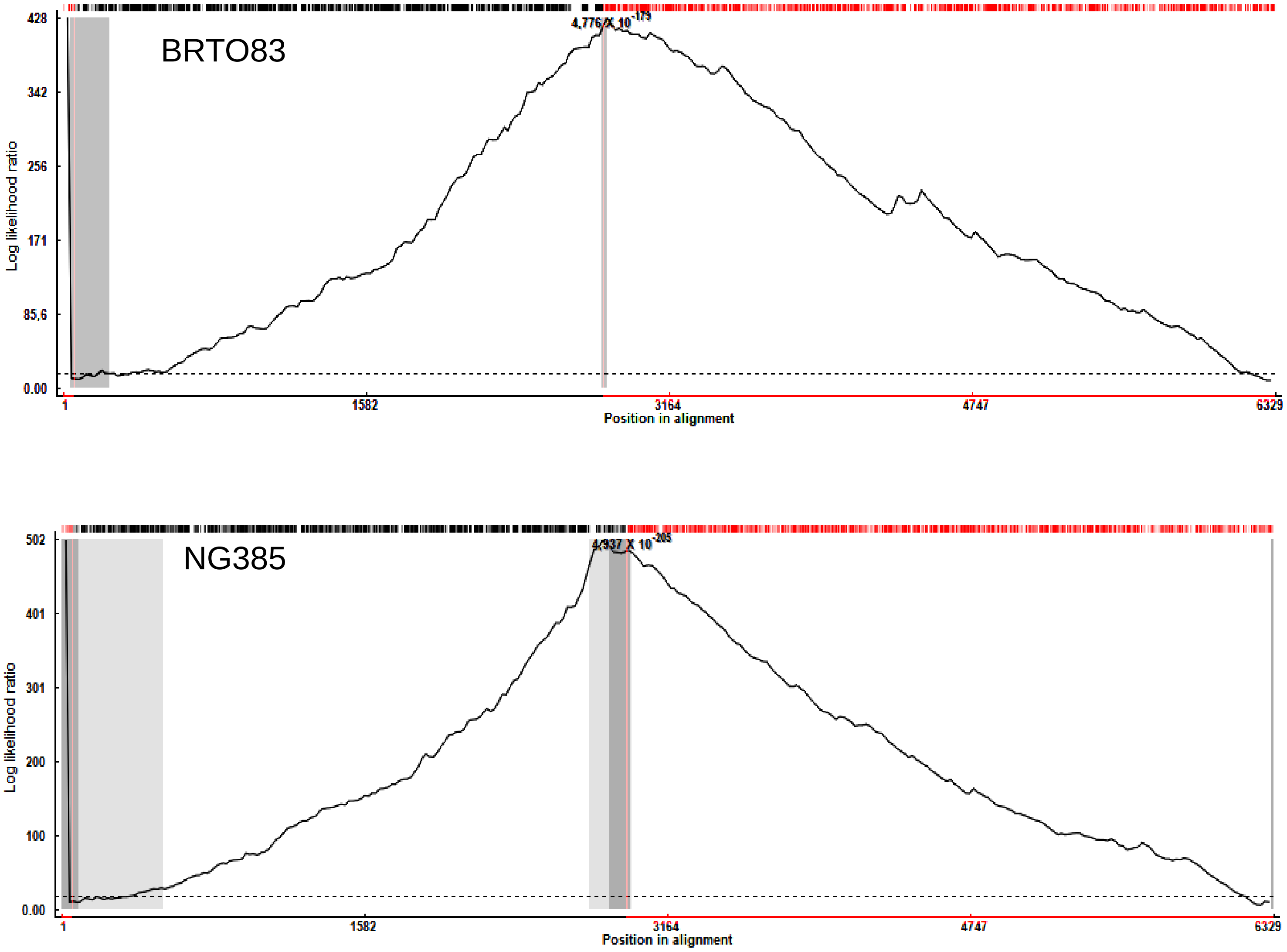

Figure 1
Recombination breakpoints detected in the polyprotein sequence of Human cosaviruses. The upper panel shows the breakpoint in the isolate BRTO83 and lower panel the breakpoint detected in the polyprotein of the isolate NG385 (JN867757). In the Y-axis values of the log likelihood are show, the x-axis shows the nucleotide positions in the polyprotein of the HuCosavirus. The plot (solid black line) indicate the point of maximum likelihood and the values above this peak is the approximate p-value. The upper dashed black and red lines indicate partitions in the alignment that presented conflicting trees. Dark gray are is the 99% confidence interval and light gray are is the 95% confidence interval for breakpoint position. Dashed line indicate the cutoff value based on Bonferroni correction test. The breakpoint location is based on the LARD method (Holmes *et al*., 1999) that detects recombination breakpoints by scanning an alignment of three sequences (a recombinant and two putative parental sequences) for the point (likelihood ratio test) in the alignment that optimally separates regions of conflicting phylogenetic signal.

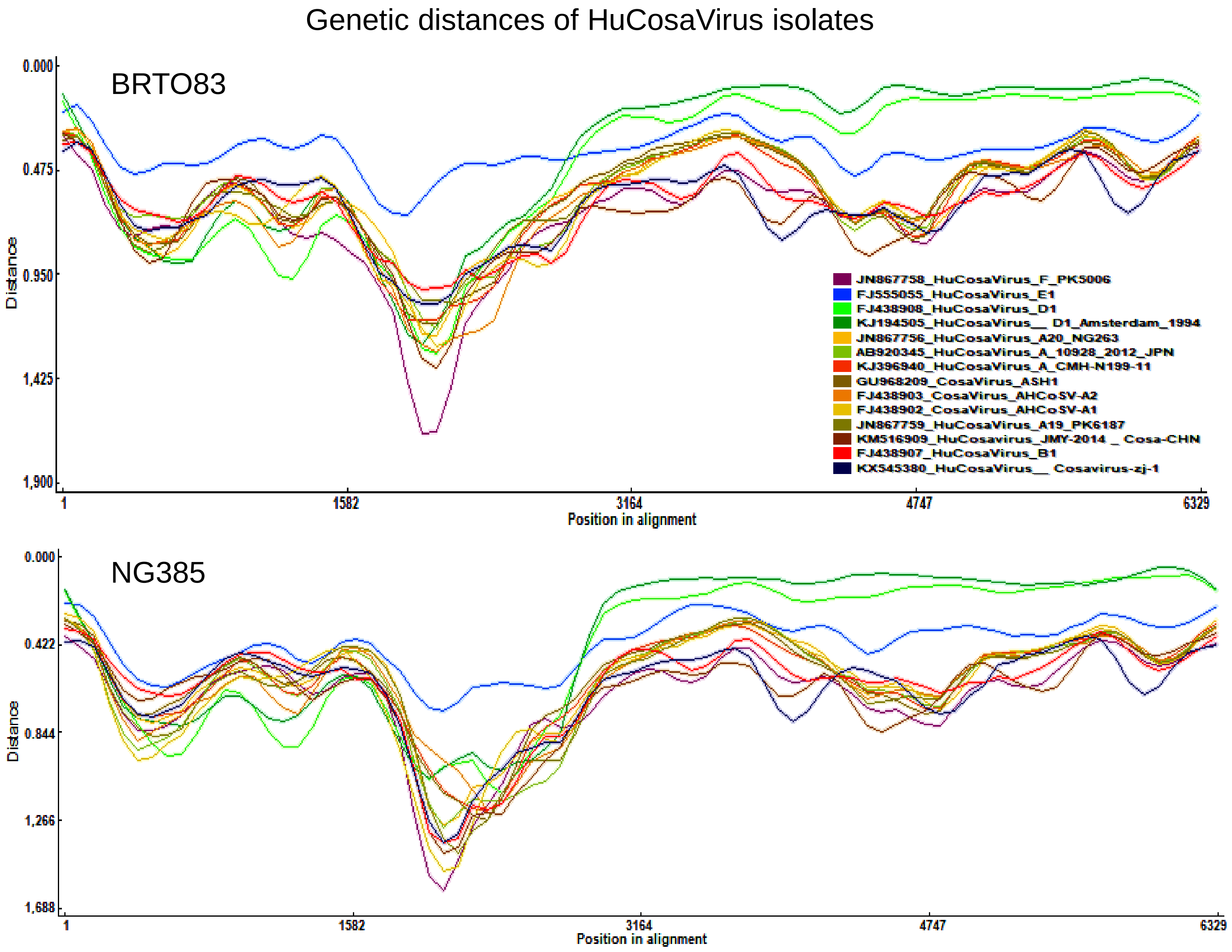

Figure 2

Genetic distances in the polyprotein sequence of Human cosaviruses.

The upper panel shows the distances between the isolate BRTO83 and the references and lower shows the distances between the isolate NG385 (JN867757) and the references. References used in this comparisons are indicated in a chart within the upper panel. The Y-axis indicate the genetic distances and the x-axis shows the nucleotide positions in the polyprotein of the HuCosavirus. Colored lines indicate the genetic distances between a certain isolate and the references. Pair-wise distances were calculated using F81 (Felsenstein J. 1984.) model and a window-based approach with window and step sizes of 200 and 30nt, respectively.

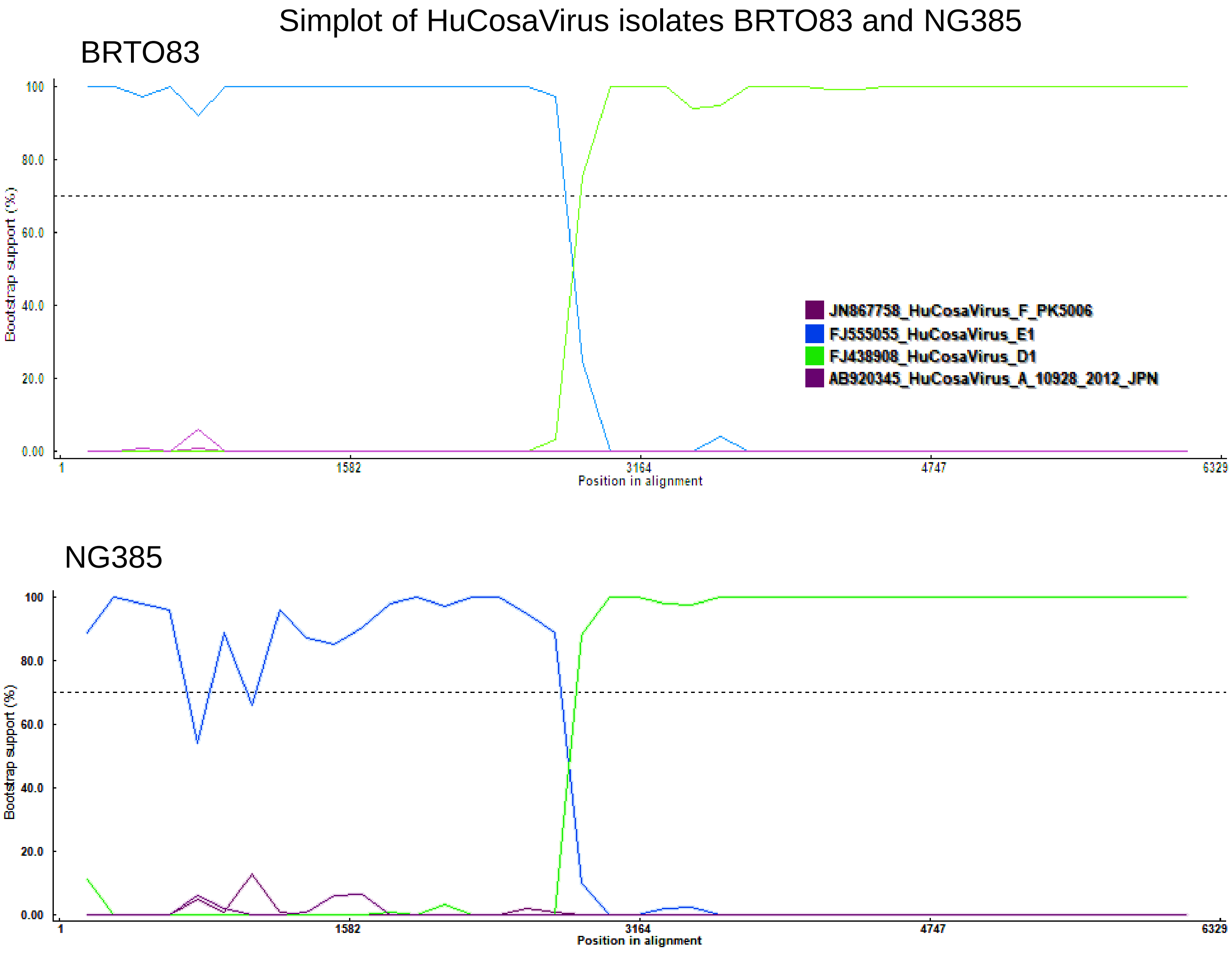

Figure 3

Mosaic pattern of the polyprotein sequence of Human cosaviruses.

The upper panel shows the mosaic pattern of the isolate BRTO83 and the lower shows the mosaic pattern of the isolate NG385 (JN867757). References used in this comparisons are indicated in a chart within the upper panel. Colored lines represent the probability (given in bootstrap value) of genomic regions to belong to a certain parental genotype. The x-axis represents the sequence length in base pairs (bp). The y-axis represents the statistical support (bootstrap) based on 500 replicates. Each plotted line refers to a certain Cosavirus genobtype (see the code color on the small panel). Vertical dashed lines represent the recombination break point on the genome region. Analyses were performed using a neighbor-joining method and Kimura 2 parameters model in windows of 150 bp sliding along sequences in increments of 60 bp. For parental references, non-recombinant sequences of genotype A, D, E, and F were used. The plot of mosaic pattern is based on the bootscan method (Salminen, et al., 1995).

Detection of Recombination;

All the analysis were performed using the software RDP v.4 (Martin et al., 2015), which utilizes a collection of methods and an excellent and detailed explanation of each method implemented in the RDP program can be found in the user’s manual (http://darwin.uvigo.es/rdp/rdp.html).

References;

Felsenstein J. 1984. Distance methods for inferring phylogenies: A justification. Evolution 38:16-24

Holmes EC, Worobey M, Rambaut A. 1999. Phylogenetic evidence for recombination in Dengue virus. Mol Biol Evol. 16:405.

Martin, D. P., Murrell, B., Golden, M., Khoosal, A. & Muhire, B. RDP4: Detection and analysis of recombination patterns in virus genomes. Virus Evol 1, vev003, doi:10.1093/ve/vev003 (2015).

Salminen MO, Carr JK, Burke DS, McCutchan FE. 1995. Identification of breakpoints in intergenotypic recombinants of HIV type 1 by BOOTSCANning. AIDS Res Hum Retroviruses 11:1423-1425.
